# “It Makes You Feel Like Someone Cares” acceptability of a financial incentive intervention for HIV viral suppression in the HPTN 065 (TLC-Plus) study

**DOI:** 10.1371/journal.pone.0170686

**Published:** 2017-02-09

**Authors:** Elizabeth Greene, Allison Pack, Jill Stanton, Victoria Shelus, Elizabeth E. Tolley, Jamilah Taylor, Wafaa M. El Sadr, Bernard M. Branson, Jason Leider, Natella Rakhmanina, Theresa Gamble

**Affiliations:** 1 Science Facilitation Department, FHI 360, Durham, North Carolina, United States of America; 2 Gillings School of Global Public Health, University of North Carolina, Chapel Hill, North Carolina, United States of America; 3 Institute for Reproductive Health, Georgetown University, Washington DC, United States of America; 4 Social and Behavioral Health Sciences, FHI 360, Durham, North Carolina, United States of America; 5 ICAP at Columbia University, Mailman School of Public Health, New York, New York, United States of America; 6 Scientific Affairs, LLC, Atlanta, Georgia, United States of America; 7 Jacobi Medical Center, New York, New York, United States of America; 8 Children’s National Health System, Washington DC, United States of America; 9 Elizabeth Glaser Pediatric AIDS Foundation, Washington DC, United States of America; London School of Hygiene and Tropical Medicine, UNITED KINGDOM

## Abstract

**Background:**

HPTN 065 (TLC-Plus) evaluated the feasibility and effectiveness of providing quarterly $70 gift card financial incentives to HIV-infected patients on antiretroviral therapy (ART) to encourage ART adherence and viral suppression, and represents the largest study to-date of a financial incentive intervention for HIV viral suppression. A post-trial qualitative substudy was undertaken to examine acceptability of the financial incentives among those receiving and implementing the intervention.

**Methods:**

Between July and October 2013, semi-structured interviews were conducted with 72 patients and 12 investigators from 14 sites; three focus groups were conducted with 12 staff from 10 sites. Qualitative data collection elicited experiences with and attitudes about the intervention, including philosophical viewpoints and implementation experiences. Transcripts were analyzed in NVivo 10. Memos and matrices were developed to explore themes from different participant group perspectives.

**Results:**

Patients, investigators, and staff found the intervention highly acceptable, primarily due to the emotional benefits gained through giving or receiving the incentive. Feeling rewarded or cared for was a main value perceived by patients; this was closely tied to the financial benefit for some. Other factors influencing acceptability for all included perceived effectiveness and health-related benefits, philosophical concerns about the use of incentives for health behavior change, and implementation issues. The termination of the incentive at the end of the study was disappointing to participants and unexpected by some, but generally accepted.

**Conclusion:**

Positive experiences with the financial incentive intervention and strategies used to facilitate implementation led to high acceptability of the intervention, despite some reluctance in principle to the use of incentives. The findings of this analysis provide encouraging evidence in support of the acceptability of a large-scale financial incentive intervention for HIV viral suppression in a clinical setting, and offer valuable lessons for future applications of similar interventions.

## Introduction

Antiretroviral therapy (ART) is critical to reducing HIV-related morbidity and mortality [1–5], as well as the risk of transmission to HIV-uninfected individuals [6]. Sustained adherence to ART can achieve the levels of viral suppression necessary to prevent HIV transmission, afford clinical benefit and avert development of resistant viral strains [7]. However, adherence remains a key challenge to realizing the full public health potential of ART in the US and worldwide [8–10].

The HPTN 065 (TLC-Plus) study (ClinicalTrials.gov number NCT01152918) aimed to evaluate the feasibility of an enhanced test, link to care, plus treat approach for HIV prevention in two communities: the Bronx, New York and Washington, D.C. The study was based on a premise derived from mathematical modelling that identifying all individuals with HIV, combined with initiation of and adherence to ART, could dramatically reduce HIV incidence in a population [11]. One component of the study tested the feasibility and effectiveness of providing financial incentives in the form of quarterly $70 gift cards for encouraging ART adherence and sustained viral suppression.

Financial incentives have been used, with varying degrees of success, to encourage uptake of a range of health-related behaviors [12], but remain an emerging area of research within the realm of HIV. Few studies have tested their efficacy in encouraging ART adherence or viral suppression [13–16], and among the latter, none have qualitatively explored their acceptability among the recipients and implementers of financial incentives interventions. Furthermore, a recent comprehensive systematic review of literature on the acceptability of a variety of health promoting financial incentives concluded that there was a dearth of empirical work on their acceptability and called for more in-depth qualitative research on this issue [17].

Between February 2011 and April 2013, 39,359 gift cards were dispensed to 9,153 patients at sites participating in the HPTN 065 study, representing the largest study to date of a financial incentive intervention for viral suppression. A distinctive feature of the HPTN 065 study was the use of a site-randomized design and aggregate surveillance data to determine the effectiveness of the intervention [18].

A qualitative substudy was undertaken to complement the parent study by systematically collecting in-depth qualitative data from a subset of patients, site investigators and staff who participated in HPTN 065, providing the opportunity for a more thorough examination of the diversity of experiences with and attitudes about the financial incentive intervention. In this analysis, we examine themes related to acceptability of financial incentives among both recipients and implementers of the intervention.

## Methods

### Financial incentives for viral suppression in the HPTN 065 study

Nineteen HIV care facilities (10 in the Bronx, 9 in D.C.) were randomized to provide the financial incentive intervention in addition to their standard of care for adherence support, and 20 HIV care facilities (10 in Bronx, 10 in D.C.) were randomized to provide standard of care only. HIV-infected patients on ART could qualify for a $70 gift card as often as quarterly for achieving or maintaining viral suppression, defined as HIV RNA <400 copies/mL. The amount of the financial incentive was determined based on extensive consultation with the study community advisory group and other stakeholders during the study design phase. The quarterly interval was selected because, at the time of the study design, quarterly blood draws to monitor CD4 cell count and viral load were recommended per guidelines for all patients except those with long-term viral suppression [19]. In order to avoid incentivizing non-adherence that might occur if patients had to newly achieve viral suppression, all patients at financial incentives sites were eligible to participate regardless of how long they had been virally suppressed.

### Qualitative substudy

Data for the qualitative substudy were collected between July and October 2013 at a subset of HIV care study sites that were randomized to the financial incentive intervention arm. Data collection and analysis occurred after the intervention had ended at all sites, but before the overall effectiveness of the incentive was assessed, so that knowledge of the effectiveness outcome would not influence participant attitudes. One-time semi-structured in-depth interviews were conducted with patients and site investigators, and focus group discussions were conducted with site staff. Demographic information was only collected from patients. Broad themes explored in the interviews and focus groups included: attitudes about the financial incentive intervention; perceived influence of the intervention on behavior, clinic attendance, and patient/provider relationships; experiences dispensing and receiving the incentives; how the gift cards were used; motivators for viral suppression and medication adherence; patients’ understanding of the purpose of the intervention; understanding of HIV viral load and its impact on health; and community awareness of the intervention.

Patient interviews and staff focus groups were conducted face-to-face by trained interviewers from diverse demographic backgrounds who were not affiliated with the parent study or substudy research team. Investigator interviews were conducted over the phone by a single member of the substudy research team trained in qualitative data collection. All interviews were conducted in English or Spanish, audio-recorded, translated into English (if necessary), and transcribed. Interviews lasted approximately 60 minutes, and focus groups, 90 minutes.

The substudy protocol was approved by a central IRB (Copernicus Group IRB) prior to commencement of data collection. Additionally, all sites recruiting patients for interviews obtained either local IRB approval (4 sites) or approval under the central IRB (10 sites). The local IRBs reviewing this study were: Albert Einstein College of Medicine of Yeshiva University IRB, Children’s National Health System IRB, and George Washington University and Medical Center IRB. Written informed consent was obtained from all patients and focus group participants prior to data collection; verbal consent was obtained from investigators prior to phone interviews. Parent/guardian consent was obtained for any participant under the age of 18, along with informed assent of the participant.

### Substudy sample

All HPTN 065 HIV care sites randomized to the financial incentive intervention were invited to participate in the qualitative sub-study. Those that agreed and were able to obtain IRB approval prior to study implementation formed the subset of sites from which participants were recruited.

For patient interviews, a non-probability, purposive, quota-based sampling strategy was employed to ensure inclusion of a heterogeneous sample of patients who had received financial incentives: participants were recruited within five subcategories based on HIV viral load values (suppressed or not) prior to and during the study period, and the date of ART initiation (before or after the intervention began) (Table 1). An initial goal of 80 patient interviews, equally distributed between the Bronx and Washington, DC and across each of the five subcategories, was estimated to be feasible and sufficient to reach saturation. In cases in which sites found it challenging to meet enrollment targets for some subcategories, slots were either reassigned or the “other” category was used as long as the patient met inclusion criteria, as the intent was to ensure a diversity of participants rather than a representative sample of the parent study.

**Table 1:** Patient subcategory distributions.

	Bronx (n)	DC (n)	TOTAL (N)
**Pre-defined Subcategories**			
ART initiated during study, VS during study (≥ 5 gift cards)	5	8	13
ART initiated during study, mixed or not VS during study (≤ 3 gift cards)	0	0	0
ART initiated prior to study, VS prior to study, VS during study (≥ 5 gift cards)	12	14	26
ART initiated prior to study, not VS prior to study, mixed or not VS during study (≤ 3 gift cards)	4	9	13
ART initiated prior to study, not VS prior to study, VS during study (≥ 5 gift cards)	0	7	7
**Other** ^a^			
≥5 gift cards	7	1	8
≤ 3 gift cards	2	3	5
**TOTAL**	**30**	**42**	**72**

^**a**^ Participant did not meet definition of pre-defined subcategories, or subcategory determination could not be made from available data.

In order to achieve a proportional distribution across subcategories and sites, participating sites, based on patient volumes, were assigned 3 to 8 participants to recruit within each subcategory. Sites were provided with standardized talking points, but determined their own recruitment strategies. To be eligible for the qualitative substudy, patients had to currently be enrolled in care at the site and had to have been eligible for the financial incentive intervention for at least 15 months of the 24-month intervention.

All investigators involved in an HPTN 065 site assigned to financial incentives for at least 12 months of the 24-month financial incentive intervention were invited by email to participate in a phone interview. All but one investigator interviewed were physicians. Focus groups included site staff members (study coordinators, nurses, social workers, research staff, and physician assistants, but not physicians) who were directly involved in implementation of HPTN 065 at a site assigned to financial incentives for at least 12 months of the 24-month financial incentive intervention. All site staff members who met these inclusion criteria were invited to participate in a focus group.

### Qualitative data analysis

All transcripts were uploaded to NVivo qualitative analysis Software Version 10.0 (QSR International Pty Ltd.) and qualitative thematic content analysis techniques were used to analyze the data, following a process of reading, coding, data display, and reduction [20]. To develop a codebook, members of the substudy research team read a subset of transcripts and coded both structural and emerging themes, reviewed coding, and refined the codebook through an iterative process until consensus and saturation were reached. The codebook was then applied to all transcripts. Approximately 20% of patient interviews, 25% of investigator interviews, and all focus group transcripts were double-coded, and two members of the research team manually reviewed results to ensure inter-coder reliability. Discrepancies were discussed amongst the research team until team members agreed on interpretation; when needed, the codebook was further clarified and transcripts recoded.

For this acceptability analysis, primary coding reports related to attitudes about the financial incentive intervention, the concept of offering financial incentives for viral suppression, experiences in giving or receiving the financial incentives and the end of the financial incentive program were extracted and further analyzed. Emergent sub-themes were coded and applied to coding reports. Where applicable, Excel matrices were used to display themes and sub-themes, and to identify patterns across data sources. Memos were developed to summarize findings within each broad theme.

## Results

Seventy-six patients from 14 sites completed the interview; 72 were included in this analysis (two did not meet eligibility criteria, one never received a financial incentive, and one appeared to be intoxicated during the interview). Additionally, 12 investigators from 14 sites completed the interview, and three focus group discussions were conducted with 12 staff members from 10 sites (Table 2). Patients ranged in age from 14 to 72 years (median age 48); 61% were male; 60% Black, 15% white, and 21% Hispanic (Table 3). The majority of patients (76%) reported an annual income of less than $20,000. (Individual-level data were not collected as part of the HPTN 065 study, and the demographic data that can be ascertained from U.S. Surveillance data for the population of patients in care at sites participating in HPTN 065 are not yet available for comparison). Patients were recruited from 4 of the 5 pre-defined subcategories; if subcategory definitions were not met, the participant was classified as “other” (Table 1). The majority of patients (75%) had received at least five gift cards over the course of the intervention.

**Table 2:** Substudy participants and sites.

	Bronx	DC	TOTAL
Patient Interviews (N) / Sites (N)	30 / 7	42 / 7	72 / 14
Investigator Interviews (N) / Sites (N)	6 / 8 ^a^	6 / 6	12 / 14
Focus Groups N (participants/sites)	2 (6 / 4)	1 (6 / 6)	3 (12 / 10)

^a^ Two investigators from the Bronx each oversaw two research sites.

**Table 3:** Patient characteristics.

	(N = 72)	(%)
Location		
Bronx	30	42%
DC	42	58%
Sex		
Female	26	36%
Male	44	61%
Transgender	2	3%
Age		
≤18	5	7%
19–25	8	11%
26–45	18	25%
>45	41	57%
Race		
Black	43	60%
White	11	15%
Other	18	25%
Ethnicity		
Hispanic	15	21%
Non-Hispanic	57	79%
Sexual Orientation		
Heterosexual	37	51%
Homosexual	28	39%
Bisexual	7	10%
Education		
Did not graduate High School/General Educational Development (HS/GED)	24	33%
HS/GED	17	24%
> HS/GED	31	43%
Personal Income in USD		
<20,000	55	76%
20,000–60,000	12	17%
>60,000	4	5%
Refused to Answer	1	1%

Nearly all patients, regardless of the number of gift cards they had received or their site, expressed an overall positive attitude regarding the financial incentive intervention. Most reported that their attitude had not changed over the course of the intervention, though a few described initial skepticism and a few had neutral attitudes about the intervention. Investigators and staff felt overall very positively about the financial incentive intervention, but expressed more mixed attitudes compared with the patients, mostly related to logistical and other challenges experienced in implementing the intervention. Three investigators noted that their attitudes regarding the financial incentive intervention had changed from skepticism in the beginning to very supportive over the course of the study.

Across patients, staff, and investigators, five main factors were identified that influenced acceptability of the financial incentive intervention: emotional benefits, financial benefits, health-related benefits, philosophical concerns and implementation issues.

### Emotional benefits

Emotional benefits were the most commonly cited reasons why patients liked the financial incentive intervention. Patients described feeling good, encouraged, appreciated, cared for, or motivated when receiving the financial incentives, and that the intervention gave them something to look forward to or be excited about. Some noted that it helped counteract the negative feelings normally associated with thinking about their HIV infection.

“And then incentives make you feel good. I mean it just makes you like ‘Okay, yay!; I got something to look forward to when I go back as long as I’m doing my medicines and eating right and stuff like that… The happy face and the handshake is good but, yeah, a [gift card] makes it even better.”

(Patient, non-Hispanic black female, 48 years old)

“Well, what I really liked about it is knowing that someone is there thinking of us and someone is there reaching out to us…. In a way you know you're already ill and stuff like that…. Most of all, it makes you feel like someone cares.”

(Patient, non-Hispanic black male, 50 years old)

Investigators and staff described overall positive opinions of the financial incentive intervention, largely attributed to emotional benefits, such as positive interactions with patients during the gift card exchange, improved morale among clinic staff and providers, and feeling good about being able to provide positive reinforcement or do something to help someone in need.

“What I liked about the study was that, when you have patients that don’t have any money to buy food or toilet paper and they would come to you with a smile on their face because they have $70 to spend on something they were not going to be able to buy, something that they needed–it’s not even things that they just wanted, it’s needed–[handing out gift cards] made me feel good.”

(Staff member)

“I think [providers] saw an excitement among their patients that they loved to see. Their patients were so happy. Whereas sometimes, a patient has to force him or herself to come in for a visit, but here they were happy to come in for a visit. They were happy to be seen, to get their blood drawn. They were reminding the doctor it was time to get their blood drawn.”

(Investigator)

Some investigators indicated that they appreciated these positive benefits so much that they would gladly implement a financial incentive intervention again in the future, whether or not financial incentives could improve viral suppression.

“My primary reason [for wanting to implement the financial incentive intervention in the future] is to provide the system of positive reinforcement … even if it doesn’t fully work. I don’t know the end results of the study. … I think it improved the dynamic of my staff in the clinic. I think we felt empowered to do something for our [patients], beyond what we do already. We do know how many of them struggle financially. We heard many positive comments.”

(Investigator)

A couple of investigators indicated that they had been opposed to the idea of offering financial incentives for viral suppression at the start of the study, but were in favor by the end because it had been such a positive experience.

“My patients thought it was an absolutely fabulous idea. … And so, frankly, over the course of the study, my attitude changed. And I became as enthusiastic as my patients were because for them this was really something that was quite exciting and they felt that they were really participating in something that was really very good.”

(Investigator)

Some staff, however, noted a negative emotional consequence in that they felt the financial incentives created a sense of entitlement in a minority of patients who became aggressive or demanding about receiving their incentive. Most of the staff who described these negative experiences still seemed to have overall positive opinions about the financial incentive intervention. However, for a few staff, these experiences seemed to be central and negatively affected acceptability for them.

“[I] definitely had a positive experience. [The financial incentive intervention] lets you get connected with the patients a little more. They definitely appreciated it. You got to know certain patients on a different level. At the same time, there were those patients … who felt entitled to get the gift card; very rude about [it] when they came to get it. Who, you know, I don’t think they cared about the viral suppression, they just wanted the money.”

(Staff member)

“I really hated the entitlement and so many people getting mad at me, cursing at me because this is their gift card. People acted like this is their paycheck, like they worked for hours to get a gift card here. Like, ‘I deserve it! I took my medication.’ And sometimes I had to step back and say, ‘This is for your health, you know that, right? It’s not just about the money.”

(Staff member)

### Financial benefits

Many patients described appreciating the financial aspects of the incentive. Most of these patients indicated that the financial incentives helped them meet their fiscal needs, and they described using the funds for necessities such as groceries, school supplies, bills, transportation, co-pays, or household and personal essentials. A number of patients also enjoyed the extra resources as a special treat, and described buying non-essential or other items either for themselves or for others as a gift, or giving the gift card itself as a gift.

“What I like about it, it was very helpful. Very helpful to me and it was very surprising like I said. … It helped me pay for my medicine. Then I got a few little personal things that females should have. … It helped me buy some groceries, you know, buy some eggs and stuff like that … Like I said, it was very helpful.”

(Patient, non-Hispanic black female, 50 years old)

“The program helped me because even though I have HIV it gave me something to look forward to every three months, buy me an outfit and make me feel good about myself.”

(Patient, non-Hispanic black female, 55 years old)

Nearly all patients, when asked, reported that they were happy with the $70 amount of the financial incentive. Although some also said they would have been happier with a higher amount, only three patients expressed that the incentive was not high enough, while two thought it was too high.

“I think it's a good amount. I think, at that level, it's a very positive incentive. I think if it was less, if it was like $25 or so, people who may not generally keep their appointments probably wouldn't do it for $25, unless they were motivated in terms of their health. But I thought $70 was very generous.”

(Patient, non-Hispanic black male, 66 years old)

“Well to be honest, we could use more, definitely use more, but $70 wasn't bad, it helped.”

(Patient, Hispanic mixed race female, 57 years old)

### Health-related benefits

A number of patients, investigators, and staff discussed the perceived effectiveness of the financial incentives in incentivizing desirable health-related behaviors or outcomes as a reason why they liked the intervention. Many of these patients did not feel that they themselves needed the financial incentive to be motivated to take their medication, but speculated that it was useful for others who have trouble with adherence.

“Like, everybody's different. For me, you don't actually have to pay me, because, like I said, I see my doctor on a regular basis anyway. My health is very important to me. But for people [that aren’t adherent] to taking medication and seeing their doctors on a regular [basis], I think that program worked for them, because it got them in a routine of going and seeing their doctors regularly.”

(Patient, non-Hispanic black female, 51 years old)

Furthermore, many of the health benefits described commonly by patients, investigators and staff were not limited to viral suppression and ART adherence, but more broadly included the perception that the card encouraged people to get in the routine of seeing their doctor and to be more proactive in their own healthcare. Some investigators and staff also felt that the financial incentive provided opportunities to encourage other, necessary, healthcare evaluations.

“It was cool. I mean, it's a good incentive thing to, you know, stay with your drugs and keep your viral load undetectable and all that stuff…it's something I think most people strive to do anyway once you get started, but it's definitely an extra incentive to, you know, get you into the doctor as well as just keeping, you know, taking your meds and everything.”

(Patient, non-Hispanic white male, 55 years old)

“We [also] got some of them to do some of their other things: their PAP smears and their EKGs and things like that. So I think, you know, I think it’s a good thing. I would want to do it again.”

(Investigator)

“None of us knew how much better our patients would do with it … we certainly knew that our patients were adhering to care much better…They were certainly much more interested in their results and getting their results back…And for a percentage of patients, … they were certainly taking their meds better, at least some percentage. So I think, you know, the providers actually really liked it.”

(Investigator)

### Philosophical concerns

When asked specifically about the concept of paying someone to achieve viral suppression, some patients had mixed reactions. While they acknowledged potential and real benefits to financial incentives, they also expressed concern that patients should be self-motivated to do what is in their own best interest. In this way, a number of patients described a philosophical conflict with the concept of offering financial incentives for viral suppression.

“People should want to just be healthy… you shouldn't want to get paid to take your medication.”

(Patient, non-Hispanic black female, 22 years old)

“I mean overall, a gift card shouldn't be … what's the word I'm looking for? A bribe, to do the right thing for your own health, but maybe some people don't have any money, so I guess it's … it's a good thing.”

(Patient, Hispanic mixed race transgender, 23 years old)

Several investigators and staff also described this philosophical conflict whereby they reported enjoying the intervention and thought it had some benefits, but similarly felt that patients should be self-motivated to stay healthy without the need for financial incentives.

“I think it was helpful because even if people take their medicine every day it’s still a positive thing to get the money…Some type of reward even though, you know, you shouldn’t get a reward for doing what you’re supposed to do.”

(Staff member)

Among those who expressed concern, some also described a philosophical conflict with the study design which required giving incentives to patients who may already be suppressed. These individuals felt that, for adherent patients, the financial incentive becomes a reward rather than an incentive, or that because resources for financial incentives may be difficult to sustain they should, therefore, be targeted to patients struggling to achieve and/or maintain their medication adherence.

“I think part of the problem was that in offering incentives to everybody, it wasn’t really incentives, it was [a] reward for many patients who [were] already suppressed.”

(Investigator)

Only a handful of patients completely disagreed with the concept of offering financial incentives for viral suppression, thinking that it was a waste of money or that it might create a divide between those who are able to achieve viral suppression versus those who are not.

No investigators or staff expressed complete opposition to the concept, though one investigator recounted initial strong resistance among providers at his clinic that quickly dissipated once the intervention started and benefits were observed.

### Implementation issues

The main challenges to acceptability for investigators and staff were related to implementation issues; the primary one being the requirement for quarterly viral load tests to qualify for financial incentives in the study. Although this schedule was intended to streamline and simplify study procedures, it did not always align with clinical care at some sites, particularly for stable patients with long-term virologic suppression who did not require such frequent monitoring [19]. This may have resulted in more crowded clinics as more patients kept their appointments or visited the clinic more frequently in order to get viral load measurements to qualify for financial incentive. Although some, particularly investigators, saw this increased engagement in care as a benefit, some also acknowledged that it increased demands on staff time and space, and sometimes interfered with clinic flow.

“We experienced significantly higher patient volume because patients were showing up for their visits and also were wanting to be rescheduled every three months, whereas otherwise they might have begged and pleaded for four month visits or six month visits or even not have scheduled right away when they left the clinic at a previous appointment. So it was a challenge for us to deal with a steadier volume.”

(Investigator)

Some staff and investigators reported that they, or the providers they worked with, felt obligated to schedule visits or lab work around the requirements for gift card eligibility rather than clinic availability, space, or patient needs, and some resented this.

“The study calls for its own needs in terms of CD4 and viral load draws. And meanwhile clinical care has sometimes different demands. …meaning that providers might not always see the need to send the CD4 and viral load on a given date. But the patient feels that if they don’t have CD4 and viral load done on those dates, they don’t get their incentives card. So there’s some times it led to conflicts between the study and the clinical care.”

(Investigator)

Other logistical challenges, though generally described as minor, may have also affected the acceptability of the financial incentive intervention for investigators and staff. Implementation required disbursing gift cards to patients, monitoring and tracking gift card inventory, ensuring safe storage of gift cards, and tracking gift card disbursement with paper forms and an electronic database. Study procedures were designed to be minimal and allow for flexible integration into workflow, but some investigators and staff noted an increased administrative burden, particularly in large clinics with a high volume of patients.

“I think that there were issues around the gift cards and making sure we kept them straight and making sure nothing got stolen. That was a big stress among the providers, and that we kept track of them properly…the operational issues were not without attention and some degree of vigilance…it was an extra layer of activity on an already burdened system, with adequate staff to do it, but still you had to implement and coordinate that, and that was a learning stress.”

(Investigator)

It was noted that logistical challenges were reported to have largely occurred during the initial phases of the study. Sites were given flexibility to develop their own processes for gift card distribution, and many described a learning curve as procedures, clinic flow, and gift card and patient tracking were improved, and attributed smooth implementation to well-trained and dedicated study staff.

“*By the end of it*, *it was just going off seamlessly*”

(Investigator).

“I think that we were very fortunate to have the young ladies that we had work on our study here. And they did a great job. And I think they learned very much how to work with the flow of the clinic, work with the providers, and work with the patients so that it really became much smoother over time.”

(Investigator)

Patients, on the other hand, largely reported no negative effect on their clinic visit experience or implementation of financial incentive intervention. Only a few noted difficulties such as longer wait times or record keeping errors related to the financial incentive intervention. Overall, implementation issues did not emerge as a theme related to acceptability of financial incentives among patients.

### Conclusion of the financial incentive intervention

The financial incentive intervention took place for a two-year duration at all sites. Patients for the most part felt neutral or slightly disappointed that they were no longer receiving the financial incentives, and some acknowledged that they had always expected the intervention to end. While disappointed to no longer be receiving gift cards, these patients understood the often provisional nature of interventions and financial resources.

“I was a little disappointed. It was a nice benefit, you know. It was … but I understand. Money runs out and things are done.”

(Patient, non-Hispanic white male, 48 years old)

Only a few patients described being distraught about the end of the intervention, indicating that they counted on it financially, or suggesting that they were only adherent because of the gift cards. One participant jokingly said

“It’s like I broke up with my best friend… I have a heartache.”

(Patient, non-Hispanic black female, 52 years old)

When describing how they were informed about the end of the financial incentive intervention, several patients used language such as “*they dropped*” *(Patient*, *non-Hispanic black male*, *58 years old)*, or “*they just kind of discontinued*” *(Patient*, *non-Hispanic*, *black male*, *34 years old)*, the intervention, indicating that there may not have been clear understanding among all patients from the start that it was only a two-year intervention as part of a research study.

A handful of patients also noted that the way they were informed about the intervention ending was not ideal.

“When I came back for my results, I thought I was gonna get [a gift card]; that’s when they told me, ‘we don’t give ‘em [out] no more’. There should have been a sign, to tell me.”

(Patient, Hispanic mixed race male, 57 years old)

Despite feelings of disappointment to varying degrees, as well as different experiences in how the end of the intervention was communicated, most patients indicated that the lack of financial incentives beyond the study would not change their reasons for taking their medication.

“I felt sad, because I got used to having [the financial incentive]. But still, I was still continuing doing my meds anyway, you know? So it wasn't that I'm taking it for that, for the gift cards, you know. So, it was just an incentive that ended.”

(Patient, Hispanic black male, 51 years old)

Investigators likewise perceived this disappointed-but-accepting sentiment among patients, noting an “*it was good while it lasted*” (*Investigator*) mentality.

“Patients are adjusting to the lack of incentives very, very well. And I haven't seen anybody who's stopped taking their meds now that they're off incentives.”

(Investigator)

Most investigators did not expect or notice any major impact, positive or negative, on their clinic or patients due to the end of the intervention, though they acknowledged that it may take more time to see the full effect, if any.

“I don't think I've seen an impact on our staff. I think they're disappointed they can't offer [the incentive] anymore, but, you know, they've got so much going on that…they've moved on. I think the patients are disappointed, like having a rich uncle come through and then he leaves…. But … we haven't seen this change long enough to know what impact that'll have.”

(Investigator)

Staff expressed more concern about the end of the program than investigators. Concerns were primarily related to anticipated or observed decreases in visit attendance, though some also speculated about potential detrimental effects on adherence or the financial stability of patients who had become dependent on incentives.

“[The end of the financial incentive intervention will have a] major impact on our clinic. Major…and it saddens us that a lot of [patients] were taking [medication] or were encouraged to take it based on receiving the gift card. It’s just sad. But we have several that are actually adherent to their medication and coming, but the visits are not as regular as they were.”

(Staff member)

## Discussion

Few studies have qualitatively explored the acceptability of financial incentives when used for health-related behavior change (particularly related to HIV), and among those that have, the populations studied have mostly been the general public, *potential* recipients, and/or *potential* implementers of a hypothetical financial incentive [12]. This study is unique in that it explored acceptability of financial incentives among actual recipients (patients) and implementers (investigators and staff) of a large-scale multi-site study about the feasibility and effectiveness of financial incentives for viral suppression among HIV-infected patients on ART. In addition, the strategic timing of the qualitative data collection and analysis—before study results on the effectiveness of the intervention were available—enabled the sharing of impressions, beliefs and attitudes based primarily on experiences with the financial incentive intervention, without the influence of the study outcome and any subsequent related discourse. The findings of this substudy reveal important insights into how a financial incentive intervention for health behavior change may be best implemented in a clinical setting. Regardless of how effective any biomedical or behavioral interventions are in the context of a clinical trial, or how great the need for them may be, their success relies in great part on user acceptance, uptake, and adherence. Qualitative studies like this one, in complement to clinical trials, are important tools for understanding the real-world contexts in which tested interventions are implemented and the factors necessary to ensure that they are acceptable to those they are intended to help.

Results from this study suggest that patients, investigators, and staff found the financial incentive intervention highly acceptable. By far the strongest facilitator of acceptability reported by both recipients and implementers was that participating in the intervention made them feel good: nearly all of those interviewed found the intervention to be a beneficial and positive experience. For many patients, this was closely tied to the receipt of a financial benefit and the additional resources it afforded, although just the positive feeling of being rewarded or cared for was a principle value for some.

In a small pilot study of financial incentives for viral suppression, Farber et al. likewise found the intervention to be acceptable to both recipients and implementers [21], but the strong emotional element identified in our analysis is novel and has not been previously reported [17, 22]. The significance of emotional benefits to implementers was unexpected and is noteworthy as emotional benefits seemed to be a key factor in mitigating implementation and other challenges that could potentially hinder acceptability. However, more research is warranted on the contribution of these emotional benefits to the mechanism of action of financial incentives in changing behavior. The shared emotional benefit also provides a new dimension to the concept of ‘fair exchange’ which has been identified by others as a key determinant in the acceptability of financial incentives. Previously this concept was conceived of as an exchange of benefits derived from the incentivized behavior, the incentive itself, and health improvement, without an emotional component on either side of the equation [17].

The findings from this study may allay concerns raised by some that offering financial incentives may compromise the provider-patient relationship that is founded on trust [23, 24]. In fact, in this study, the financial incentive intervention generally seemed to foster a stronger and more positive relationship. However, in rare circumstances, interactions with aggressive or “entitled” patients created negative impressions for staff members. These incidents, though not frequently noted, nonetheless indicate that financial incentives do have the potential for a negative effect on provider/patient interactions. If not undertaken in an environment of mutual respect and open communication, financial incentives may potentially be detrimental to patient-provider relationships. Setting clear expectations may help to reduce the potential for these negative interactions, and having a plan in place to help staff prevent or manage incidents may offset any damaging consequences.

The findings of this study also revealed complex conflicting feelings regarding offering financial incentives for health behaviors, highlighting a ‘philosophical conflict’ whereby the financial incentive intervention was acceptable and even gratifying, but associated with reported concerns, even among recipients themselves, that one should not have to ‘pay people to do what is good for them.’ This sentiment is not unique and has been cited in the literature, reflecting a common perception of wider societal beliefs with regard to the use of health-promoting incentives [17, 22, 25, 26]. Our findings suggest that some individuals who might be theoretically opposed may change their thinking after a tangible and positive experience.

We found that the specific incentive that was offered in HPTN 065 –a $70 gift card–was highly acceptable to the participants in this study. However, the quarterly disbursement schedule, while desirable for patients, proved to be at times disruptive for providers as it was more frequent than expected or needed for many patients. These findings support previous studies which have found that financial incentive acceptability may depend on the type, format, size and scheduling of the incentives [17]. Further research is needed to investigate the optimal frequency of financial incentives for effectiveness, feasibility, and acceptability.

Sustainability is a common concern in the context of financial incentive interventions, with some fears that such rewards may create a beneficial effect that does not persist due to dependence on a financial resource that may only be temporary [23, 25, 27]. While these concerns were echoed by some investigators and staff interviewed, for patients, the discontinuation of the financial incentives at the end of the study was disappointing but generally accepted. In some cases, the planned discontinuation could have been communicated more effectively. While there was largely no indication that the end of this intervention would have detrimental effects on medication adherence behaviors, it should be noted that, at the time of this publication, it is not known whether or not patients who received financial incentives were able to maintain viral suppression after the end of the intervention.

Previous studies, mostly conducted amongst the general population and which involved hypothetical financial incentives, have found that the most consistent and strongest factor influencing their acceptability was the demonstrated effectiveness and cost-effectiveness of the incentive [17, 22, 28]. Likewise, we found that the perceived or observed effectiveness of the financial incentives in this study seemed to be a factor for some; however, our findings differed from previous studies in that we noted other, perhaps even stronger factors influencing acceptability by the participants, while cost-effectiveness specifically was not indicated as a factor. Our findings suggest that high acceptability may be possible in the context of unknown effectiveness of financial incentives.

This study had several limitations. Participants were drawn from a convenience sample and, thus, are not representative of all those participating in the HPTN 065 study or the wider population of HIV-infected individuals. The intent was to examine a diversity of experiences, however, it is possible that those who did not have positive experiences with the intervention may have been less likely to agree to participate in the study. This may be supported by the fact that the study sample was comprised mostly of patients who had received five or more gift cards. Further, patients may have been overly motivated to receive one final financial incentive in the form of compensation for their participation in the interviews, and thus may have been more likely to report positive opinions. It also should be noted that the majority of the substudy sample was low income and thus may have been more beneficially affected by the incentives than a higher-income population. Additionally, the findings may not be applicable to settings outside of the United States or even in different domestic contexts. Investigators were interviewed for the substudy by a member of the HPTN 065 protocol team with whom they may have had a professional relationship during the course of the study, which presents the possibility of social desirability bias. However, our review of interview transcripts provides no evidence for this given the forthright and sometimes critical nature of some investigator reflections.

## Conclusion

HPTN 065 represents the largest study to-date of the use of financial incentives among HIV-infected patients receiving ART to maintain viral suppression. The success and long-term sustainability of financial incentive interventions are dependent on their acceptability by both recipients of the incentive as well as by those implementing the intervention. Positive experiences with the financial incentive intervention in the HPTN 065 study and strategies used to overcome implementation challenges facilitated high acceptability of the intervention, despite some resistance in principle to the use of financial incentives to influence health behaviors. The findings of this analysis provide encouraging evidence for the acceptability of a large-scale financial incentive intervention for viral suppression in a clinical setting, and offer valuable lessons for the future use of financial incentives.
